# Supplementary material for: Crystal Structures of Fusion Cores from CCoV-HuPn-2018 and SADS-CoV
Source: Viruses. 2024 Feb 9;16(2):272. doi: 10.3390/v16020272 (PMC10893436; doi:10.3390/v16020272)
Supplement: Supplementary file 1 [file viruses-16-00272-s001.zip › viruses-2772229-supplementary.pdf]

Supplementary Materials:

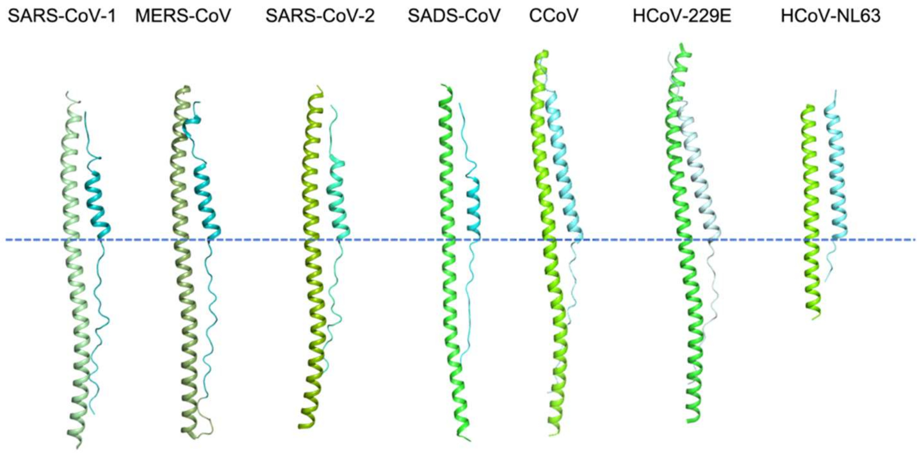

Figure S1. HR2 monomers from representative coronaviruses. SARS-CoV-1 (PDB entry: 2BEZ), MERS-CoV (PDB entry: 4NJL), SARS-CoV-2 (PDB entry: 6LXT), HCoV-229E (PDB entry: 5YL9), HCoV-NL63 (PDB entry: 2IEQ) and two our structures.

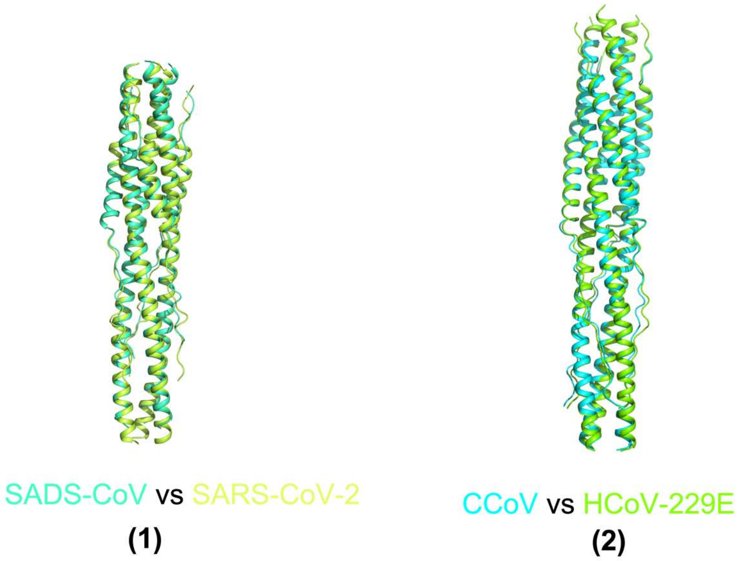

Figure S2. (lemon) Fusion cores (**1**) and CCoV-HuPn-2018 (cyan) and HCoV-229E (chartreuse) fusion cores (**2**), using SARS-CoV-2 (PDB entry: 6LXT) and HCoV-229E (PDB entry: 5YL9) as reference, respectively.

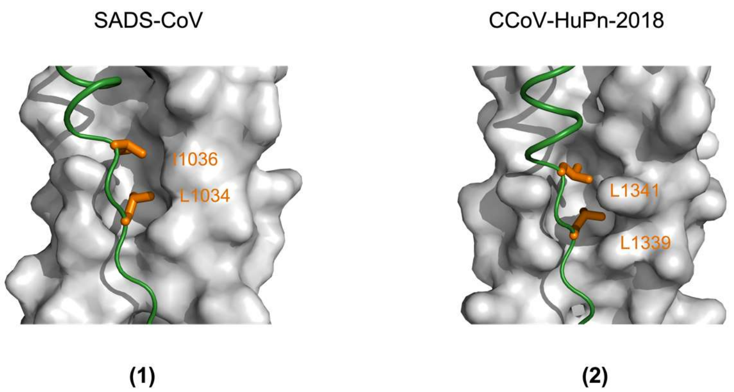

Figure S3. Trimer with residues L1034 and I1036 (SADS-CoV) (**1**) or L1339 and L1341 (CCoV-HuPn-2018) (**2**) from HR2 pointing toward it.

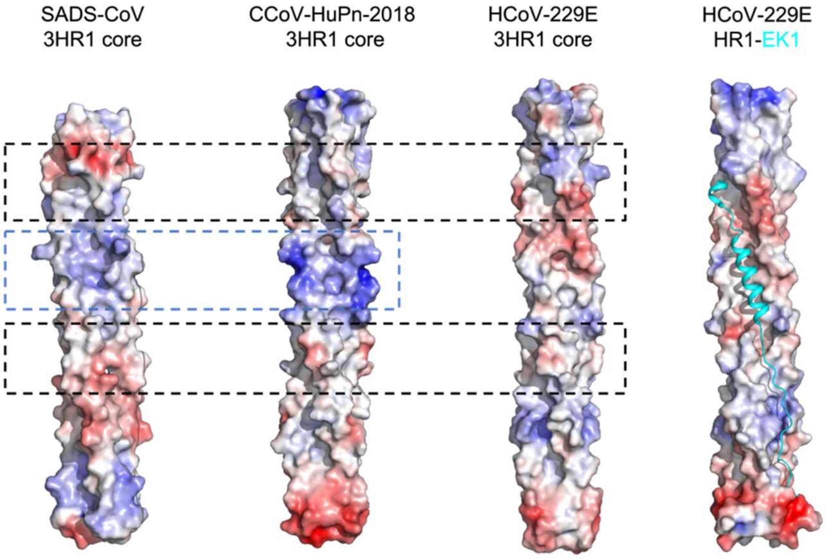

Figure S4. HR1 core from SADS-CoV, CCoV-HuPn-2018 and HCoV-229E. The black dashed boxes depict the regions on different 3HR1 cores where the electrostatic surface potentials are similar among three α-HCoVs, while the blue dashed boxes depict the regions on 3HR1 cores where the electrostatic surface potentials are similar in two α-HCoVs. Structure of HR1-EK1 6-HB is shown on the right panel as electrostatic surface (3HR1) and ribbon (EK1, cyan).
